# Effects of the KEIGAAF intervention on the BMI z-score and energy balance-related behaviors of primary school-aged children

**DOI:** 10.1186/s12966-020-01012-8

**Published:** 2020-08-17

**Authors:** Sacha R. B. Verjans-Janssen, Sanne M. P. L. Gerards, Stef P. J. Kremers, Steven B. Vos, Maria W. J. Jansen, Dave H. H. Van Kann

**Affiliations:** 1 Department of Health Promotion, NUTRIM School of Nutrition and Translational Research in Metabolism, Maastricht University, 6229 HA Maastricht, The Netherlands; 2 Department of Industrial Design, Eindhoven University of Technology, 5612 AZ Eindhoven, The Netherlands; 3 School of Sport Studies, Fontys University of Applied Sciences, 5644 HZ Eindhoven, The Netherlands; 4 Academic Collaborative Center for Public Health, Public Health Service South-Limburg, 6400 AA Heerlen, The Netherlands; 5 Department of Health Services Research, Maastricht University, CAPHRI Care and Public Health Research Institute, 6229 GT Maastricht, The Netherlands

**Keywords:** Health promoting schools, BMI, Physical activity, Nutrition, Children, Intervention, Primary school

## Abstract

The aim of the current study was to evaluate the one- and two-year effectiveness of the KEIGAAF intervention, a school-based mutual adaptation intervention, on the BMI z-score (primary outcome), and energy balance-related behaviors (secondary outcomes) of children aged 7–10 years.

A quasi-experimental study was conducted including eight intervention schools and three control schools located in low socioeconomic neighborhoods in the Netherlands. Baseline measurements were conducted in March and April 2017 and repeated after one and 2 years. Data were collected on children’s BMI z-score, sedentary behavior (SB), physical activity (PA) behavior, and nutrition behavior through the use of anthropometric measurements, accelerometers, and questionnaires, respectively. All data were supplemented with demographics, and weather conditions data was added to the PA data. Based on the comprehensiveness of implemented physical activities, intervention schools were divided into schools having a comprehensive PA approach and schools having a less comprehensive approach. Intervention effects on continuous outcomes were analyzed using multiple linear mixed models and on binary outcome measures using generalized estimating equations. Intervention and control schools were compared, as well as comprehensive PA schools, less comprehensive PA schools, and control schools. Effect sizes (Cohen’s d) were calculated.

In total, 523 children participated. Children were on average 8.5 years old and 54% were girls. After 2 years, intervention children’s BMI z-score decreased (*B* = -0.05, 95% CI -0.11;0.01) significantly compared to the control group (*B* = 0.20, 95% CI 0.09;0.31). Additionally, the intervention prevented an age-related decline in moderate-to-vigorous PA (MVPA) (%MVPA: *B* = 0.95, 95% CI 0.13;1.76). Negative intervention effects were seen on sugar-sweetened beverages and water consumption at school, due to larger favorable changes in the control group compared to the intervention group. After 2 years, the comprehensive PA schools showed more favorable effects on BMI z-score, SB, and MVPA compared to the other two conditions.

This study shows that the KEIGAAF intervention is effective in improving children’s MVPA during school days and BMI z-score, especially in vulnerable children. Additionally, we advocate the implementation of a comprehensive approach to promote a healthy weight status, to stimulate children’s PA levels, and to prevent children from spending excessive time on sedentary behaviors.

Trial registration

Netherlands Trial Register, NTR6716 (NL6528), Registered 27 June 2017 – retrospectively registered.

## Introduction

Childhood overweight and obesity are related to an increased risk of premature mortality and cardiometabolic morbidity in adulthood [1]. In recent decades, childhood overweight and obesity prevalence has increased globally, but the trend has plateaued since around 2000 in many high-income countries [2, 3]. However, prevalence is still high and intervention is warranted. In the Netherlands, about 13.1% of children (aged 4–12 years) were overweight, of whom 3.3% were obese in 2017 [4]. Overweight is the result of an imbalance in physical activity and dietary intake. Only half (55.5%) of Dutch children (aged 4–12 years) met the physical activity (PA) recommendations of 60 min of moderate-to-vigorous PA per day and muscle-strengthening activities three times per week [4]. Dietary behaviors of Dutch children are similarly suboptimal. Only two in five Dutch children (aged 4–12 years) consumed sufficient amounts of fruit and vegetables [4]. Dutch children frequently consume foods and beverages that are high in calories (due to high levels of sugar and fat) and low in nutritional value, e.g., about 17% of the daily energy intake is from energy-dense snacks and drinks [5–7]. Of the beverages consumed by children aged 4 to 8 years, 45% contain sugar [7]. These unhealthy PA and dietary behaviors are particularly prominent in children of low socioeconomic position families [8–10].

Given that school-aged children spend a significant amount of their time at school, the school is a popular intervention setting. Schools can improve PA levels of children by offering opportunities for children to engage in PA throughout the school day (e.g., during recess, through physical education) [11]. Although Dutch primary schools do not provide lunches, have no vending machines or school canteens [12], schools can still improve children’s nutrition behavior. They can do this by implementing food policies concerning the home-packed lunches and drinks (mostly consisting of sandwiches, water, milk or a sugar-sweetened beverage, and sometimes candy or biscuits) and providing healthy foods (e.g., a policy promoting water or providing fruit) [11, 13, 14]. Evidence concerning the effectiveness of school-based PA and nutrition interventions on children’s energy balance-related behaviors and Body Mass Index is inconclusive [15–25]. Explanations for the mixed results could be that each school context is unique with different needs, resources and perspectives on PA and healthy nutrition promotion [26]. To be feasible, acceptable and to reduce the risk of problems during implementation, interventions should fit this unique context [27] and local adaptation should be allowed during intervention implementation [28]. Additionally, to maximize effects of school-based interventions, they should be comprehensive. A comprehensive school health-promoting approach is an approach that promotes PA and healthy nutrition behavior by educating schoolchildren, providing a stimulating physical and social environment, and by engaging the parents and the wider community [29, 30]. Comprehensive school health promotion interventions have the potential to reduce children’s BMI, increase PA, improve fruit and vegetable consumption and water intake [24, 31], and reduce the intake of unhealthy foods and drinks [24].

We implemented a school-based PA and nutrition intervention with a high level of flexibility in the design to enhance contextual fit [32]. Ideally, the intervention resulted in a comprehensive PA and nutrition-promoting approach [32]. The KEIGAAF (a Dutch acronym for ‘Chances in Eindhoven for a family-based approach by Fontys’) intervention was implemented in primary schools located in low socioeconomic neighborhoods in the Netherlands. In this study, the main objective was to evaluate the effects of the intervention on the Body Mass Index (BMI) z-score (primary outcome measure), sedentary behavior (SB), PA behavior, and nutrition behavior (secondary outcomes) of children aged 7–10 years after one and 2 years. Secondly, we investigated whether schools with a comprehensive PA and nutrition-promoting approach showed better results on the primary and secondary outcomes. We hypothesized that the intervention shows desirable effects on BMI z-score, SB, PA, and nutrition behavior (i.e., breakfast, fruit and vegetables, snack, sugar-sweetened beverages, and water consumption) after 2 years, and that applying a comprehensive PA and nutrition-promoting approach would result in more beneficial outcomes, compared to a less comprehensive approach.

## Methods

### Study design

A quasi-experimental study was conducted to evaluate the effects of the KEIGAAF intervention on children’s BMI z-score and energy balance-related behaviors. Eleven primary schools – eight intervention schools and three control schools – located in low socioeconomic neighborhoods in the south of the Netherlands participated in the study. The control schools were located in a different municipality, which resembled the intervention region based on level of urbanization and socioeconomic status of the schools’ neighborhoods. The study design, recruitment of study participants, and data collection tools used have been described in detail in the protocol paper of Verjans-Janssen et al. [32]. The medical ethics committee of Maastricht University Medical Centre provided ethical approval for the study (METC163027, national number: NL58554.068.16) and the study is registered in the Netherlands Trial Register (NTR6716).

### The KEIGAAF intervention

The KEIGAAF intervention was implemented between April 2016 and June 2019. The general aim of the intervention was to promote PA behavior and healthy nutrition behavior among the children. To do this, a mutual adaptation approach was used in which top-down principles and influences interacted with bottom-up development and implementation of PA and healthy nutrition-promoting activities. A steering committee of health behavioral experts and representatives of local organizations (a school board, a sports support organization, a social work organization, the municipal health service organization, and a youth work organization) provided basic intervention principles (top-down) to local working groups who developed local activity plans and implemented these activities (bottom-up). In short, these intervention principles were: (1) each school formed an interdisciplinary working group, consisting of school staff, local (health) professionals, parents, and a health promotion advisor (the composition differed per school); (2) the working groups developed and implemented the intervention according to the needs of the children and the possibilities within the community; (3) the intervention was aimed at improving PA and nutrition behavior; and (4) the working groups decided which behavior to target first, to what extent, and what order. The eight working groups were supported by the same health promotion advisor during the entire intervention period, except for one. In total, there were four health promotion advisors. In this mutual adaptation approach, the local context and ownership was honored while basic intervention principles and broader system influences were acknowledged [32–34]. This process of mutual adaptation differed per school [34]. More details on the design of this approach can be found elsewhere [32, 34].

The health promotion advisors, and health behavioral experts from research institutes, advised the schools in implementing a comprehensive approach of PA and healthy nutrition-promoting activities. A comprehensive approach is an approach in which practice and policies are aligned and when PA and healthy nutrition behavior are promoted by educating children, providing a supportive social and physical environment and stimulating healthy energy balance-related behaviors before and after school time (i.e., by involving parents and the wider community) [29, 30, 35]. Implementation of the intervention in the schools was a dynamic process consisting of many feedback loops: the process was improved continuously based on evaluations, advice of the health promotion advisor and the feedback of research data. This dynamic process resulted in different intervention activities per school. Examples of implemented activities were the use of new PA equipment during school recess, provision of water bottles to children, implementation of monthly after-school sports activities, and applying a policy concerning healthy birthday treats at school. Intervention activities were new or strengthened existing activities. A list of implemented PA and healthy nutrition-promoting activities of the schools can be found in Additional file 1. Implementation of the KEIGAAF intervention has been described in detail elsewhere [34].

### Study participants

At baseline, all primary school children in grades four to six (aged 7 to 10 years) were eligible for inclusion. No additional inclusion or exclusion criteria were defined. The primary researcher informed the children orally about the study and provided an information letter to their parents. Parents could ask the primary researcher questions during planned school meetings. For a child to participate, two parents had to provide written consent. Children and parents participated in the baseline measurements (T0) conducted in March and April 2017, and the follow-up measurements after one (T1) and 2 years (T2), i.e., March/April 2018 and 2019. Collecting data in the same period reduced the risk of seasonal variation in BMI and PA behavior [36, 37].

### Measurements

The same data were collected for the intervention group and the control group. Data were collected on the children’s BMI z-score as primary outcome measure and SB, PA behavior and nutrition behavior as secondary outcome measures.

#### BMI z-score

To measure children’s BMI z-score, trained research assistants assessed children’s weight and height using a measurement protocol. Children were weighed and measured during a physical education lesson. Children wore light sports clothes and shoes were taken off before measurements were made. A stadiometer (Seca 213, Hamburg, Germany) was used to measure standing height with an accuracy of 1 mm, and a digital weighing scale (Seca 803, Hamburg, Germany) was used to measure the child’s weight to the nearest 0.1 kg. Weight and height were used to calculate BMI. BMI was recoded into BMI z-score standardized for age and gender, based on a Dutch reference population [38]. International cut-off points were used to define if children were underweight (BMI z ≤ − 1.65), had a normal weight (− 1.65 < BMI z < 1.04) or were overweight (1.04 < BMI z < 1.64) or obese (BMI z ≥ 1.65) [39].

Parents also filled in their child’s height in centimeters (no decimals) and weight in kilograms (no decimals) in the parent questionnaire. These data were used to impute missing baseline data on a child’s BMI z-score. This imputed BMI z-score was used as covariate in the analyses on the effectiveness on PA behavior and dietary behavior, but not as outcome measure. Data were imputed for seven children (five intervention and two control).

#### Sedentary and physical activity behavior

The ActiGraph GT3X+ accelerometer (ActiGraph, Pensacola, FL, USA) was used to measure children’s SB and PA behavior. Children wore the accelerometer strapped around their waist for seven consecutive days during waking hours. The accelerometer was removed when performing activities involving water (e.g., swimming and showering). Accelerations were recorded at a sampling frequency of 30 Hz using 10s time intervals. ActiLife version 6.13.3 was used to filter accelerometer data. Wear time was validated using Choi’s classification criteria [40]. Additionally, a valid wear day was defined as providing at least 480 min of valid wear time per day between 06.00 AM and 11.00 PM [41]. For this study, only data recorded on schooldays were included. The first wear day was excluded to reduce bias due to reactivity to the accelerometer measurement [42]. Additionally, non-regular school days (such as festive days where the children attended school only half a day) were excluded to ensure that the data reflected PA behavior on a regular school day. Children were included in the analysis when they had at least two school days with valid accelerometer data at the time of measurement. To classify the accelerometer data into SB, light PA (LPA) and moderate-to-vigorous PA (MVPA), Evenson’s cut-off points were used (SB: ≤ 100 counts per minute (CPM), LPA: 101 < CPM < 2295 and MVPA: ≥ 2296 CPM) [43]. The data were aggregated into average SB, LPA, and MVPA per child. Additionally, the vector magnitude CPM (the sum of counts over the three axes) was used as outcome measure. The data on SB and PA were supplemented with data on weather conditions during the measurement periods to adjust for potential weather influences on PA behavior in the analyses [44]. For this, data on the average temperature, and total hours of sunshine and precipitation between 06.00 AM and 11.00 PM of the Royal Dutch Meteorological Institute were used. These data were also aggregated into daily averages within this time period.

#### Nutrition behavior

Children’s nutrition behavior at school was assessed using a child questionnaire, while children’s daily nutrition behavior was assessed using a parent questionnaire. Children filled in a paper questionnaire individually at school during school hours. Children filled in whether they had consumed breakfast in the morning (on the current school day), and whether they had consumed fruit, vegetables, candy, cookies, savory snacks, sugar-containing beverages, energy drinks, sports drinks, or water during the previous school day. The answer options were yes/no and the questions were based on the Local and National Youth Monitor of the Netherlands, but were made understandable for children (i.e., by using simple language and adding images) [45]. The items fruit consumption and vegetable consumption were combined into the variable ‘fruit or vegetables’, which was given a score of ‘yes’ when fruit and/or vegetables were consumed on a regular school day and ‘no’ when none of these items was consumed. The same was done for the variable ‘sugar-containing beverages’ (consisting of the items daily consumption of sugar-containing beverages, energy drinks, and sports drinks) and the variable ‘candy, cookies or savory snacks’.

One parent was asked to fill in a paper questionnaire at home. The parent reported on the child’s average nutrition behavior during a normal week in the previous month. For this, items from a validated food frequency questionnaire were used [46]. For this study, data on the consumption of fruit, vegetables (raw and cooked), candy (e.g., sweet, licorice, candy bars), savory snacks (e.g., cheese, crisps), sugar-sweetened beverages, fruit juice, sweet milk drinks, and water were included. The food frequency was measured using answer options ranging from zero to 7 days (i.e., the number of days per week). The daily amounts of fruit and vegetables were measured in natural units: pieces and serving spoons, respectively. One serving spoon of vegetables was considered 50 g. The average daily consumption of fruit and vegetables was calculated. For this, the frequency and amount were multiplied and divided by seven. The nutrition data were not normally distributed, therefore the data were recoded into binary outcome measures based on the frequencies and amounts of fruit and vegetables consumed and the frequencies of the consumption of snacks (including candy and savory snacks), sugar-sweetened beverages (including sugar-sweetened beverages, fruit juice, and sweet milk) and water (including tea without sugar). Fruit and vegetables were recoded into adherence to the recommendations for fruit and vegetables, respectively (yes/no). Snacks, sugar-sweetened beverages, and water were recoded into daily consumption (no: ≤ 6 days per week, yes: 7 days per week). To assess the adherence to the recommendations for fruit and vegetables, the national recommendations of the Dutch Nutrition Centre were used: 1.5 pieces of fruit per day for children ≤ 8 years and two pieces for children ≥ 9 years, and 100–150 g vegetables per day for children ≤ 8 years and 150–200 g for children ≥ 9 years [47]. There are no national recommendations for snacks, sugar-sweetened beverages, and water, other than to ‘limit the consumption of snacks and sugar-sweetened beverages and consume 1-1.5 liters of liquids per day (ideally water, but milk is also allowed)’ [47].

#### Socio-demographic characteristics

Socio-demographic characteristics of the child and the parent were assessed using the questionnaires. Children reported their date of birth (to calculate their age), their gender, the country of birth of both parents (to determine ethnicity), and the zip code of their home address. Based on the definition of Statistics Netherlands of ethnicity, the child’s ethnicity was considered non-Western when at least one parent was born in a non-Western country (a country in Africa, Latin America, Asia (excluding Indonesia and Japan), or Turkey) [48]. The zip code of the home address was used to define the socioeconomic status (SES)-score of the child’s residential neighborhood from the Netherlands Institute for Social Research. This score is based on the educational level, income, and employment status of the residents. A high score indicates a high SES in that neighborhood [49].

The parent reported their year of birth (to calculate age), educational level, family situation (living together, single, other), height and weight (to calculate BMI) and zip code of the home address for this study. Missing child data on the home zip code were supplemented by using the data on zip code provided by the parent. Educational level was recoded into three categories based on the International Standard Classification of Education 2011 [50]: no or primary educational level (no education or primary school), secondary educational level (pre-vocational school, secondary education, or lower vocational education), and tertiary educational level (higher vocational education or university degree). Parents’ reported height (in centimeters, no decimals) and weight (in kilograms, no decimals) were used to calculate their BMI and define their weight status (i.e., BMI < 20: underweight, BMI 20–25: normal weight, BMI 25–30: overweight, and BMI > 30: obese).

### Comprehensiveness of PA and nutrition-promoting approach

**Fig. 1:**
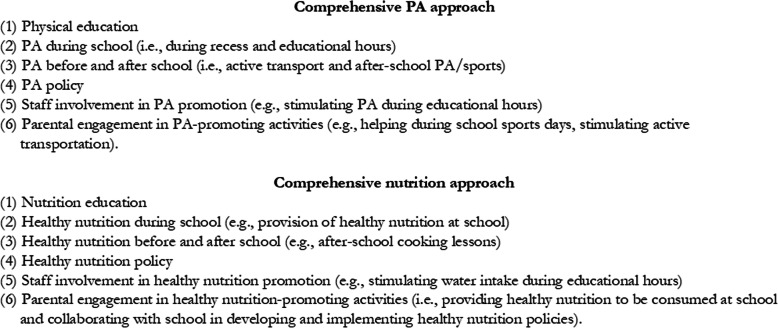
Components of a comprehensive PA and healthy nutrition approach (based on [29])

Data on the intervention school’s PA promotion and healthy nutrition-promoting activities implemented in the intervention period were obtained by conducting timeline sessions and using an online school scan [32, 34]. A list of PA and healthy nutrition-promoting activities of the schools at the end of the intervention period can be found in Additional file 1. The activities were divided into the main categories of a comprehensive health-promoting approach: (1) PA/healthy nutrition education; (2) PA/healthy nutrition during school; (3) PA/healthy nutrition before and after school; (4) PA/healthy nutrition policy; (5) staff involvement in PA/healthy nutrition promotion; and (6) parental engagement in PA/healthy nutrition-promoting activities (Fig. 1) [29]. Schools were considered comprehensive (yes/no) when activities were implemented in all categories, there was coherence between practice and policies, and when the message was spread consistently via different channels (i.e., by the teachers, other school staff, the children, and their parents) in and outside the school environment [35]. Besides the use of timeline sessions (an evaluation method to assess implementation) and an online school scan, minutes of the working group meetings and participatory observations were used to decide on the level of comprehensiveness concerning PA and nutrition promotion [32, 34]. None of the intervention schools were comprehensive concerning nutrition promotion. For example, only three schools implemented healthy nutrition-promoting activities before and after school and these activities had a low reach. Additionally, there was low variation between the intervention schools regarding the nutrition-promoting activities implemented during the intervention period (Additional file 1). Therefore, it was not possible to divide the intervention schools into groups based on their level of comprehensiveness concerning nutrition promotion.

### Statistical analysis

Descriptive statistics were performed to compare the baseline characteristics of the children and parents in the intervention and control group. T-tests and Chi-square tests were conducted to compare the groups on continuous and categorical baseline demographic characteristics respectively, and the primary and secondary outcome measures. When the assumption of normality or the assumption of equal variances between groups was not met, the Welch’s test and the Mann Whitney U test were used, respectively. We analyzed the intervention effects on continuous outcomes using multiple linear mixed models and on binary outcome measures using generalized estimating equations (GEE), and accounted for the repeated measures within individuals. Mixed models are able to handle missing data in a longitudinal dataset without needing to perform multiple imputations [51]. Based on the results of the Likelihood Ratio Test, a random intercept for school was added to the model. This was only necessary for the PA classifications, i.e., SB, LPA and MVPA. For each outcome variable, two models were created: (1) effects over time for each group were analyzed by including a time variable (baseline, 1 year, and 2 years) as fixed factor in the model; (2) the differences over time between the groups were analyzed by adding the group variable (intervention/control) and the interaction between the group variable and time as fixed factors to Model 1. BMI z-score was adjusted for child ethnicity (Western/non-Western) and residential socioeconomic status (SES). BMI z-score is the preferred measure because it allows for comparison between children of different ages and between boys and girls. However, it is advised to also analyze effects on BMI to enhance comparison of intervention studies [31]. Therefore, we performed the same analyses with BMI as outcome measure. Analyses were adjusted for child age, gender, ethnicity (Western/non-Western), and residential SES. PA and nutrition outcomes were adjusted for child ethnicity (Western/non-Western), residential SES, child age, gender (boy/girl) and BMI z-score at baseline [5, 52, 53]. Additionally, the PA outcomes were adjusted for weather conditions (temperature, sunshine, and precipitation) [44]. For the GEE analyses, the logit link function and an exchangeable correlation matrix was applied. To analyze intervention effects for the comprehensive PA schools and the less comprehensive PA schools separately, we conducted the same analyses but now three groups were compared in separate analyses: (1) the comprehensive PA schools versus the control schools; (2) the less comprehensive PA schools versus the control schools; and (3) the comprehensive PA schools versus the less comprehensive PA schools. These groups were compared on all outcome measures. IBM SPSS Statistics for Windows, version 25.0 (IBM Corp, Armonk, NY, USA) was used for the statistical analyses. *P* values <.05 were considered statistically significant. A power calculation was conducted for the sample size at the beginning of the study [32]. For the given sample size, the smallest detectable difference in the primary outcome measure (i.e., BMI z-score) after 2 years of intervention ranges between 0.38 and 0.44 when the power is 80%, indicating a moderate effect size [32]. To interpret the magnitude of the effects, effect sizes (Cohen’s d) were calculated by dividing the estimated between-group difference by the pooled standard deviation of the outcomes [54]. Lipsey cut-off points were used to interpret the effect sizes as small (≤ 0.32), moderate (0.33–0.55), and large (≥ 0.56) effects [55].

## Results

### Study participants

**Fig. 2:**
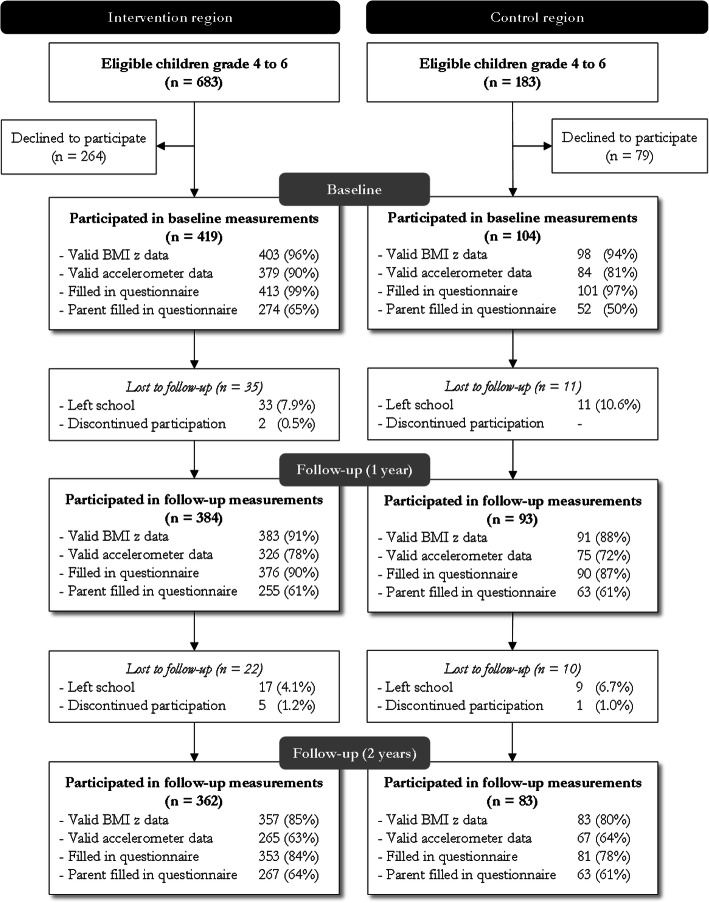
Flow of the participants. *Note.* Percentages are based on participants at baseline.

Of the eligible children, 523 children (60%) participated in the study at baseline (Fig. 2). Valid anthropometric data were obtained for 501 children (96%) at baseline, 474 children (91%) at the first follow-up measurement (after 1 year), and 440 children (84%) at the final follow-up measurement (after 2 years). At baseline, first follow-up, and final follow-up, 463, 401, and 332 children (89, 77, and 64%) provided valid accelerometer data, respectively. Of the participating parents, 326, 318 and 330 (62, 61 and 63%) filled in the parent questionnaire and 514, 466, and 434 children (98, 89, 83%) filled in the child questionnaire, at baseline, first follow-up, and final follow-up, respectively. There was a total loss of 78 children in the study (14% in the intervention group and 20% in the control group, non-significant). Of these, eight children (10.3%) discontinued participation and 70 children left school. Dropout analyses revealed that children who discontinued participation (*N* = 8) had a higher BMI z-score at baseline (*M* = 1.36, *SD* = 0.48) compared to the retained study participants (*M* = 1.06, *SD* = 0.05) (*t*(436) = − 2.59, *p* = .01).

### Demographics

At baseline, children were on average 8.5 years old and 54% were girls (Table 1). Most children had a normal weight (74%). Compared to the control region, relatively more non-Western children participated in the intervention region (*χ*^*2*^ (1, *N* = 520) = 11.38, *p* = .001). Additionally, the parents who had filled in the questionnaire at baseline were on average significantly older (*t*(400) = 3.73, *p* < .001) and higher educated in the intervention region compared to the control region (*χ*^*2*^ (2, *N* = 405) = 16.85, *p* < .001). There were no significant differences in other socio-demographic child and parent variables between children attending intervention and control schools.

**Table 1:** Characteristics of the study population

	Intervention group (***N*** = 419)		Control group (***N*** = 104)		Total (***N*** = 523)	
	M (SD)	N (%)^**b**^	M (SD)	N (%)^**b**^	M (SD)	N (%)^**b**^
*Child characteristics at baseline*						
Age (years)	8.47 (1.05)		8.68 (1.07)		8.51 (1.06)	
Gender						
Boys		191 (45.6)		49 (47.1)		240 (45.9)
Girls		228 (54.4)		55 (52.9)		283 (54.1)
Ethnicity^a^						
Western		223 (53.5)		74 (71.8)		297 (57.1)
Non-Western		194 (46.5)		29 (28.2)		223 (42.9)
BMI z-score	0.23 (1.09)		0.22 (0.97)		0.23 (1.06)	
Weight status						
Underweight		13 (3.3)		3 (3.1)		16 (3.2)
Normal weight		293 (73.3)		76 (77.6)		369 (74.1)
Overweight		55 (13.8)		11 (11.2)		66 (13.3)
Obese		39 (9.8)		8 (8.2)		47 (9.4)
*Parent characteristics at baseline*						
Age (years)^a^	39.15 (5.58)		36.45 (5.91)		38.64 (5.74)	
Educational level^a^						
Low		80 (24.2)		34 (45.9)		114 (28.1)
Middle		124 (37.5)		26 (35.1)		150 (37.0)
High		127 (38.4)		14 (18.9)		141 (34.8)
Family situation						
Living together		272 (81.9)		55 (73.3)		327 (80.3)
Single		60 (18.1)		20 (26.7)		80 (19.7)
BMI (kg/m^2^)	25.03 (4.19)		26.01 (4.95)		25.19 (4.33)	
Weight status						
Normal weight		145 (57.8)		28 (56.0)		173 (57.5)
Overweight		106 (42.2)		22 (44.0)		128 (42.5)

*Note. M* Mean, *SD* Standard deviation, *N* number of participants, *BMI* Body Mass Index

^a^Significantly different at baseline compared with the control group

^b^Total N of categorical variables can vary due to missing data

### Intervention effects on BMI z-score

**Fig. 3:**
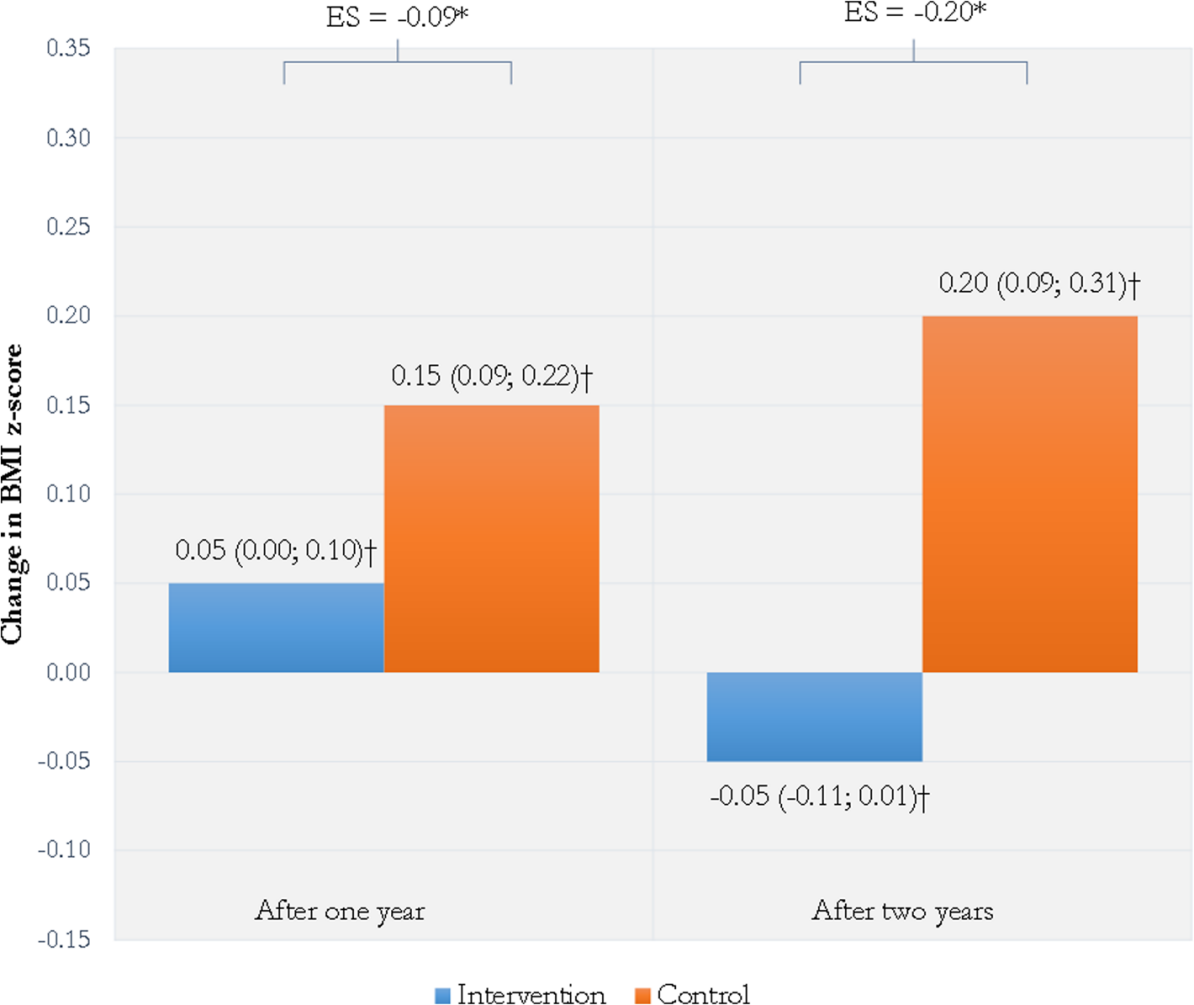
Change in children’s BMI z-score after one and two years. *Note.* BMI z-score is adjusted for child age and gender. Repeated measures linear mixed model analyses were adjusted for clustering of data within persons, child ethnicity (Western/non-Western) and residential socioeconomic status score. ES = Effect size (Cohen’s d). * Significant difference (1 year: *B* = − 0.11, 95% CI -0.21; 0.00, *p* = .04, 2 years: *B* = − 0.25, 95% CI -0.38; − 0.12, *p* < .001). † Numbers shown are unstandardized beta-coefficient and 95% confidence interval of linear mixed model (B (95% CI))

The intervention group and the control group did not differ significantly in BMI z-score at baseline (Table 1). After 1 year, the intervention group and control group both increased in BMI z-score (*p* = .05 and *p* < .001, respectively) (Fig. 3). This increase was significantly smaller for the intervention group (*B* = − 0.11, 95% CI -0.21; 0.00, *p* = 0.04, ES − 0.09). After 2 years, the intervention group decreased in BMI z-score and the control group increased (*p* = .08 and *p* = .001, respectively). This difference was significantly different (*B* = − 0.25, 95% CI -0.38; − 0.12, *p* < .001) and the effect size was small (ES = − 0.20). Comparable results were found when analyzing intervention effects on BMI (1 year: *B* = − 0.33, 95% CI -0.58; − 0.08, *p* = .01, ES − 0.10 and 2 years: *B* = − 0.43, 95% CI -0.79; − 0.07, *p* = .02, ES − 0.13).

### Intervention effects on physical activity behavior

At baseline, the control group performed on average relatively more MVPA (8.6%, 66.2 ± 29.0 min) compared to the intervention group (7.8%, 60.6 ± 22.5 min) (*p* = .06). Both the intervention and the control group showed a significant increase in SB during school days and a significant decrease in LPA during school days after one and 2 years (Table 2). Favorable intervention effects were found on MVPA during school days after 2 years. Although the intervention group showed a significant decrease in MVPA (observed mean difference: − 7.0 ± 20.7 min), the control group showed a significantly larger decrease (observed mean difference: − 14.4 ± 28.7 min) (*p* = .02). The effect size was small (ES = 0.22).

**Table 2:** Change in sedentary and physical activity behavior during school days of intervention and control groups after one and 2 years (Model 1) and one- and two-year intervention effects (Model 2)

		Model 1								Model 2		
		Intervention group (***N*** = 419)				Control group (***N*** = 104)				Intervention group vs. control group		
		N	Mean (SD)^**a**^	B (95% CI)^**b**^	***p***	N	Mean (SD)^**a**^	B (95% CI)^**b**^	***p***	B (95% CI)^**b**^	***p***	ES
%SB	T0	379	61.34 (6.58)			84	60.03 (7.11)					
	T1	326	64.46 (6.39)	**2.23 (1.58; 2.89)**	**0.00**	75	62.66 (6.95)	**2.24 (0.49; 3.99)**	**0.01**	−0.14 (−1.64; 1.36)	0.85	−0.01
	T2	265	66.65 (6.40)	**4.89 (4.16; 5.62)**	**0.00**	67	66.39 (6.83)	**4.45 (2.47; 6.43)**	**0.00**	−0.41 (−2.15; 1.33)	0.64	−0.04
%LPA	T0	379	30.88 (5.07)			84	31.34 (5.36)					
	T1	326	28.40 (4.92)	**−1.82 (−2.34; −1.31)**	**0.00**	75	29.11 (5.13)	**−2.09 (−3.46; −0.73)**	**0.00**	0.18 (−1.00; 1.36)	0.76	0.02
	T2	265	26.53 (4.77)	**−4.19 (−4.76; − 3.63)**	**0.00**	67	27.01 (5.27)	**−2.72 (−4.19; −1.26)**	**0.00**	−0.57 (−1.91; 0.77)	0.40	−0.07
%MVPA	T0	379	7.78 (2.89)			84	8.63 (3.78)					
	T1	326	7.14 (2.59)	**−0.36 (−0.67; −0.06)**	**0.02**	75	8.23 (3.18)	−0.01 (− 0.74; 0.73)	0.98	− 0.06 (− 0.76; 0.64)	0.86	−0.01
	T2	265	6.82 (2.82)	**−0.69 (−1.02; − 0.36)**	**0.00**	67	6.60 (2.34)	**−1.51 (−2.58; − 0.44)**	**0.01**	**0.95 (0.13; 1.76)**	**0.02**	**0.22**
CPM	T0	379	1153.89 (288.75)^c^			84	1270.87 (489.96)					
	T1	326	1040.72 (259.40)	**−80.65 (− 109.43; −51.88)**	**0.00**	75	1131.00 (323.44)	−97.79 (− 207.32; 11.45)	0.08	35.87 (−40.17; 111.90)	0.35	0.07
	T2	265	972.08 (283.38)	**− 161.14 (− 193.73; − 128.55)**	**0.00**	67	926.21 (227.56)	**− 278.18 (− 410.66; − 145.69)**	**0.00**	**130.52 (43.21; 217.83)**	**0.00**	**0.26**

*Note.* Model 1: predictor variable is time / Model 2: predictor variable is time*condition (reference group = control group). Analyses were conducted using mixed model analysis with adjustment for clustering of data within persons and adjustment for clustering of data at school level

*SB* Sedentary behavior, *LPA* Light physical activity behavior, *MVPA* moderate-to-vigorous physical activity behavior, *CMP* counts per minute, *N* number of participants, *T0* baseline measurement, *T1* measurement at year one, *T2* measurement at year 2, *SD* standard deviation, *CI* Confidence Interval, *B* unstandardized beta coefficient, *p* = *p* value.

Bold numbers are significant at *p* < .05.

^a^Mean is the observed SB, LPA, MVPA and CPM of participants with data

^b^Unstandardized beta coefficient of linear mixed model adjusted for child age, gender, ethnicity, and BMI z-score at baseline, residential socioeconomic status score at baseline and weather conditions (i.e., sunshine, temperature, and precipitation)

^c^Significantly different at baseline compared with the control group, analyzed using the Welch’s test

### Intervention effects on nutrition behavior at school

Child-reported data showed that children in intervention schools consumed significantly more fruit and vegetables at school than children in control schools at baseline (89.3% versus 75.2%, *χ*^*2*^ (1, *N* = 513) = 13.80, *p* < .001) (Table 3). No statistically significant intervention effects were found on the percentage of children consuming breakfast before school, the percentage of children consuming fruit or vegetables at school and the percentage of children consuming candy, cookies, or snacks at school after one and 2 years. After one and 2 years, statistically significant negative intervention effects were found on the percentage of children consuming sugar-sweetened beverages at school (OR = 4.86 and OR = 5.68, respectively) and water at school (OR = 0.08 and OR = 0.39, respectively). This was due to a smaller decrease and smaller increase in the percentage of children in the intervention group consuming sugar-sweetened beverages and water, respectively, compared to the control group.

**Table 3:** Child-reported change in the percentage of children consuming breakfast before or specific foods and drinks at school for the intervention and control groups after one and 2 years (Model 1) and one- and two-year intervention effects (Model 2)

		Model 1								Model 2	
		Intervention group (***N*** = 419)				Control group (***N*** = 104)				Intervention group vs control group	
		N	Obs %^**a**^	OR (95% CI)^**b**^	***p***	N	Obs %^**a**^	OR (95% CI)^**b**^	***p***	OR (95% CI)^**b**^	***p***
Breakfast (%yes)	T0	413	93.0			101	87.1				
	T1	376	90.2	0.70 (0.42; 1.14)	0.15	90	76.7	**0.46 (0.24; 0.91)**	**0.03**	1.52 (0.63; 3.69)	0.35
	T2	353	89.0	0.62 (0.38; 1.02)	0.06	81	75.3	**0.38 (0.19; 0.75)**	**0.01**	1.68 (0.69; 4.08)	0.26
Fruit or vegetables (%yes)	T0	412	89.3^c^			101	75.2				
	T1	375	89.9	1.43 (0.94; 2.18)	0.09	90	75.6	0.95 (0.50; 1.80)	0.86	1.45 (0.70; 3.12)	0.31
	T2	352	90.9	**1.60 (1.03; 2.49)**	**0.04**	81	74.1	0.92 (0.48; 1.79)	0.81	1.70 (0.78; 3.70)	0.18
Candy, cookies or snacks (%yes)	T0	412	43.2			101	42.6				
	T1	376	42.0	0.96 (0.74; 1.25)	0.76	90	35.6	0.76 (0.43; 1.32)	0.33	1.30 (0.71; 2.37)	0.40
	T2	353	37.1	0.77 (0.59; 1.01)	0.06	81	38.3	0.87 (0.49; 1.54)	0.64	0.89 (0.48; 1.66)	0.72
Sugar-sweetened beverages (%yes)	T0	412	54.1			101	47.5				
	T1	376	50.0	0.87 (0.68; 1.11)	0.24	90	13.3	**0.17 (0.09; 0.33)**	**0.00**	**4.86 (2.44; 9.68)**	**0.00**
	T2	352	38.1	**0.52 (0.40; 0.67)**	**0.00**	81	7.4	**0.09 (0.04; 0.20)**	**0.00**	**5.68 (2.37; 13.59)**	**0.00**
Water (%yes)	T0	408	57.6			101	59.4				
	T1	376	63.0	1.18 (0.93; 1.51)	0.18	90	95.6	**15.56 (5.75; 42.12)**	**0.00**	**0.08 (0.03; 0.22)**	**0.00**
	T2	352	71.6	**1.85 (1.43; 2.41)**	**0.00**	81	86.4	**5.17 (2.52; 10.58)**	**0.00**	**0.39 (0.19; 0.79)**	**0.01**

*Note.* Model 1: predictor variable is time (reference group = baseline measurement) / Model 2: predictor variable is time*condition (reference group = control group*baseline measurement). Analyses were conducted using GEE analysis with adjustment for clustering of data within persons. *N* number of participants, *I* intervention group, *C* control group, *T0* baseline measurement, *T1* measurement at year one, *T2* measurement at year two, *Obs %* observed percentage, *OR* Odds Ratio, *CI* Confidence Interval, *p* = *p* value

Bold numbers are significant at *p* < .05.

^a^Observed percentage of children that consumed breakfast on the morning of data collection and that consumed the food/drink the previous day at school

^b^Odds ratio of GEE model adjusted for child age, gender, ethnicity, BMI z-score at baseline, and residential socioeconomic status score at baseline

^c^Significantly different at baseline compared with the control group, analyzed using the Chi-square test

### Intervention effects on daily nutrition behavior

No statistically significant intervention effects were found on the parent-reported percentage of children adhering to the fruit recommendation, the percentage of children adhering to the vegetable recommendation, the percentage of children consuming snacks daily, and the percentage of children consuming water daily after 1 year. Statistically significant favorable intervention effects were found on the percentage of children consuming sugar-sweetened beverages daily after 1 year (OR = 0.45), due to a statistically significant decrease in the percentage of children consuming sugar-sweetened beverages after 1 year in the intervention group (OR = 0.67) and a non-significant increase in the control group (OR = 1.54) (Table 4). No statistically significant intervention effects were found after 2 years on daily nutrition behavior.

**Table 4:** Parent-reported change in the percentage of children adhering to or daily consuming specific foods and drinks for the intervention and control groups after one and 2 years (Model 1) and one- and two-year intervention effects (Model 2)

		Model 1								Model 2	
		Intervention group (***N*** = 419)				Control group (***N*** = 104)				Intervention group vs. control group	
		N	Obs %^**a**^	OR (95% CI)^**b**^	***p***	N	Obs %^**a**^	OR (95% CI)^**b**^	***p***	OR (95% CI)^**b**^	***p***
Adherence fruit recommendation (%yes)	T0	263	38.4			48	27.1				
	T1	242	42.6	1.08 (0.79; 1.48)	0.63	57	22.8	0.81 (0.32; 2.05)	0.66	1.34 (0.56; 3.18)	0.51
	T2	252	35.3	0.80 (0.58; 1.10)	0.17	58	20.7	0.64 (0.25; 1.64)	0.35	1.22 (0.50; 2.96)	0.67
Adherence vegetable recommendation (%yes)	T0	250	26.0			52	23.1				
	T1	233	22.7	0.76 (0.51; 1.14)	0.19	58	12.1	0.44 (0.15; 1.27)	0.13	1.81 (0.61; 5.37)	0.29
	T2	239	17.3	**0.54 (0.35; 0.83)**	**0.01**	54	3.7	**0.12 (0.02; 0.56)**	**0.01**	4.49 (0.99; 20.29)	0.05
Daily consumption of snacks (%yes)	T0	274	46.7			52	50.0				
	T1	255	31.0	**0.56 (0.42; 0.74)**	**0.00**	61	44.3	0.90 (0.47; 1.74)	0.75	0.60 (0.31; 1.17)	0.13
	T2	264	34.5	**0.70 (0.53; 0.93)**	**0.01**	63	28.6	**0.42 (0.21; 0.85)**	**0.02**	1.59 (0.79; 3.17)	0.19
Daily consumption of sugar-sweetened beverages (%yes)	T0	270	55.9			51	52.9				
	T1	249	43.8	**0.67 (0.49; 0.89)**	**0.01**	62	59.7	1.54 (0.77; 3.10)	0.22	**0.45 (0.22; 0.92)**	**0.03**
	T2	265	37.4	**0.53 (0.39; 0.71)**	**0.00**	63	42.9	0.80 (0.40; 1.60)	0.53	0.68 (0.33; 1.40)	0.30
Daily consumption of water (%yes)	T0	273	67.4			52	61.5				
	T1	251	70.5	1.22 (0.90; 1.66)	0.20	62	72.6	**2.00 (1.04; 3.85)**	**0.04**	0.62 (0.30; 1.27)	0.19
	T2	267	71.2	1.28 (0.95; 1.74)	0.11	62	71.0	1.85 (0.96; 3.59)	0.07	0.72 (0.35; 1.47)	0.36

*Note.* Model 1: predictor variable is time (reference group = baseline measurement) / Model 2: predictor variable is time*condition (reference group = control group*baseline measurement). Analyses were conducted using GEE analysis with adjustment for clustering of data within persons. *I* Intervention group, *C* control group, *N* number of participants, *T0* baseline measurement, *T1* measurement at year one, *T2* measurement at year two, *Obs %* observed percentage, *OR* Odds Ratio, *CI* Confidence Interval, *p* = *p* value

Bold numbers are significant at *p* < .05.

^a^Observed percentage of children adhering to the recommendation (for fruit and vegetables) or daily consuming the food/drink (i.e., snacks, sugar-sweetened beverages, and water)

^b^Odds ratio of GEE model adjusted for child age, gender, ethnicity, BMI z-score at baseline, and residential socioeconomic status score at baseline

### Differences between the comprehensive PA schools and the less comprehensive PA schools

**Fig. 4:**
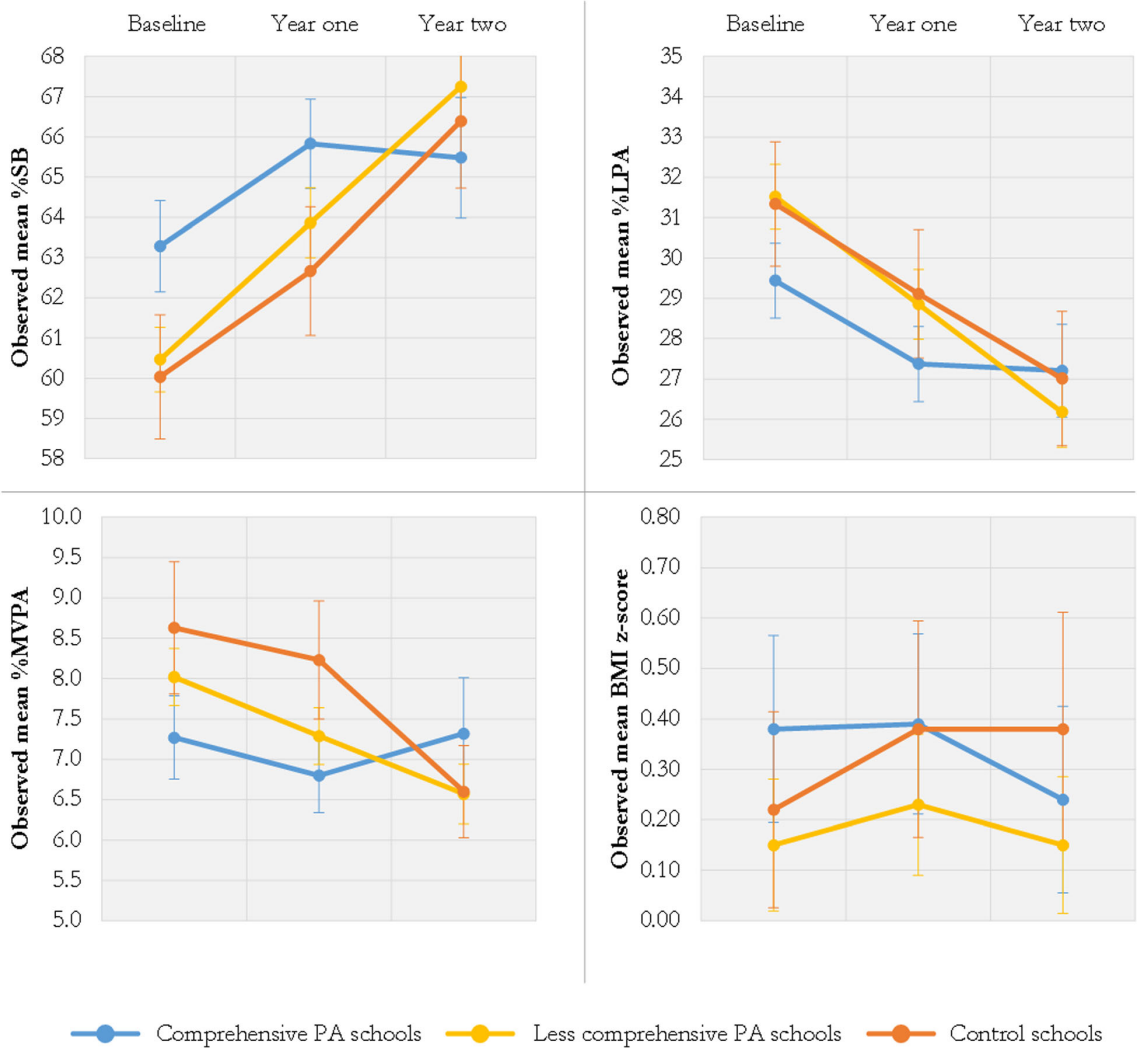
Children’s %SB, %LPA, %MVPA and BMI z-score at baseline, year one and year two for comprehensive PA schools, less comprehensive PA schools, and control schools

The comprehensive PA schools (*N* = 4) differed significantly from the less comprehensive PA schools (*N* = 4) and the control schools in effects on BMI z-score and PA behavior (Fig. 4). The comprehensive PA schools showed a reduction in BMI z-score after 2 years, while the less comprehensive PA schools resumed to baseline levels, and the control schools showed an increase. These differences were statistically significant and effect sizes were small (ES = − 0.06 and ES = − 0.21). Additionally, children exposed to a comprehensive PA-promoting approach showed a smaller increase in SB and had the same levels of MVPA during school days compared to their levels at baseline, while the children in the other conditions showed larger increases in SB and a decrease in MVPA during school days over time (Fig. 4). These differences were statistically significant and the effect sizes were small-to-moderate (Additional file 2). The comprehensive PA schools also showed statistically significant favorable changes on LPA during school days when compared to the less comprehensive PA schools (*B* = 2.09, 95% CI 0.83; 3.36, *p* < .001, ES 0.19).

Additionally, the comprehensive PA schools showed some statistically significant favorable intervention effects on nutrition intake in comparison to the less comprehensive PA schools. The comprehensive PA schools showed significantly favorable effects on children’s candy, cookies and snack intake, and sugar-sweetened beverages consumption at school compared to the less comprehensive PA schools after 1 year (OR = 0.50 and OR = 0.57, respectively). These effects were less or diminished after 2 years (Additional file 2). After 2 years, the comprehensive PA schools showed significantly favorable effects on adherence to the fruit recommendation compared to the less comprehensive schools (OR = 2.04). In contrast, the less comprehensive PA schools had significantly more favorable effects on adherence to the vegetable recommendation compared to the control schools (OR = 5.42) (Additional file 2).

## Discussion

The aim of the current study was to evaluate the effects of the KEIGAAF intervention on the BMI z-score, SB, PA behavior, and nutrition behavior of children aged 7–10 years after one and 2 years. Favorable intervention effects were found on children’s BMI z-score after one and 2 years and on children’s MVPA during school days after 2 years. After 2 years, children in the intervention group had a lower BMI z-score, while BMI z-score increased for the children in the control condition. A decline in MVPA during school days was prevented in the intervention group compared to the control group. After 1 year, favorable intervention effects were seen on children’s daily consumption of sugar-sweetened beverages, while negative intervention effects were found on children’s consumption of water and sugar-sweetened beverages at school. The positive intervention effect on the daily consumption of sugar-sweetened beverages was not present after 2 years. Contrastingly, after 2 years, the negative intervention effects on children’s consumption of water and sugar-sweetened beverages at school persisted. We found that these favorable two-year effects on BMI z-score and MVPA mainly occurred in the schools that implemented a comprehensive PA-promoting approach. Additionally, these comprehensive PA schools showed favorable intervention effects on children’s SB and LPA.

The working groups of three of the four comprehensive PA schools saw a clear need for improvement at the start of the intervention. They perceived that children’s PA and nutrition behavior needed improvement and that change concerning PA and healthy nutrition promotion at school was necessary [34]. These perceptions of the working groups corresponded to the actual behavior of the children. Children of comprehensive PA schools were significantly more sedentary and engaged in less light physical activity at baseline compared to the children of the less comprehensive PA schools and the children of the control schools (Additional file 2). Baseline MVPA levels of children of comprehensive PA schools only differed significantly from children of the control group and not from the less comprehensive PA schools. BMI z-score, however, did not differ between conditions. Looking at the demographics of the comprehensive PA schools versus the less comprehensive PA schools, it was found that a significantly smaller proportion of the parents in the comprehensive PA schools were highly educated (25.9 and 44.4% high education; respectively). Besides, significantly more children with a non-Western background were attending schools that applied a comprehensive PA approach (56.7% versus 41.3%). Given the presence of socioeconomic and ethnic inequalities in child health [56], it seems that the schools with the most vulnerable population and in need of behavioral improvement succeeded in implementing a comprehensive approach, which resulted in the most favorable intervention effects on BMI z-score and MVPA.

These results underline the importance of a long-term intervention [31, 57]. For the schools to implement a comprehensive approach, at least 1 year of preparation and 2 years of implementation were needed [34]. Activities were mainly implemented towards the end of the first year of implementation and during the second year of implementation, explaining why (the most favorable) intervention effects on BMI z-score and MVPA were mainly found after 2 years. Unfortunately, this time period was too short for schools to implement a comprehensive nutrition approach. Schools experienced children’s nutrition behavior as being more difficult to change at school than physical activity [34]. This is because collaboration between school and parents is important in creating a healthy nutrition-promoting environment, but this collaboration is difficult to achieve [58]. Parents have a big influence on children’s nutrition consumption, also at school, because Dutch children bring home-packed lunches and drinks to school [12]. To change children’s nutrition consumption at school, schools can set rules concerning what is allowed to be consumed at school [14], but (expected) resistance from parents and staff toward these rules inhibits implementation [34].

This difficulty in implementing nutrition-related activities explains why the intervention schools were not able to achieve favorable intervention results on children’s drinking behavior compared to the control schools. During the two-year study period, the control schools were actively engaged in the Dutch EPODE approach [59, 60]. This approach is an intersectoral community approach aimed at reducing childhood obesity by promoting a healthy lifestyle in children [59, 60], which has proven to be effective in improving children’s nutrition behavior [61]. The approach has a strong focus on the consumption of water [62], explaining the large effects found in the control group on sugar-sweetened beverages and water consumption.

We found that the KEIGAAF intervention led to a deviation from the negative trend in MVPA during school days, as well as BMI z-score observed in the control schools and shown globally [63, 64]. Children attending comprehensive PA schools actually showed an increase in MVPA of approximately 30 min per week from year one to year 2 on school days. Additional mixed model analyses revealed that intervention effects on PA were limited to the school day and not present on SB or PA when a whole week was taken into account (data not shown). This was in line with our expectation, since the intervention was mainly implemented in the school environment, but could also imply a potential compensation effect during the weekends. However, whole week analyses were slightly underpowered due to less adherence to the wear protocol during weekends and therefore these results should be interpreted cautiously. Yet, the effects on MVPA during school days were promising, because initially the PA levels declined over time. Annual declines in total PA and MVPA in children are seen worldwide and already occur at the age of 8 years and peak around the age of nine [63, 65], which was the age at which we intervened. The effect on BMI z-score is important, given the high numbers of overweight and obesity in our population at baseline. Even though the effect size was small (ES = − 0.20), effects can have a great impact at population level when implemented at a larger scale [66]. For example, the mutual adaptation approach can be embedded in national school health promoting initiatives in the Netherlands, such as the Healthy School program [67].

There are few studies on comprehensive school health promotion as compared to the number of studies on classroom-only intervention studies [35]. Our comprehensive school health promotion intervention consisted of an intervention that was flexible in content, locally appropriate, and that allowed adaptation of top-down principles to local needs and adaptation of local implementation to top-down provided support (i.e., mutual adaptation) [34]. To our knowledge, only the AS! BC model in Canada [68], the APPLE project in Canada [69, 70]; the APPLES intervention in the United Kingdom [71], and the Active Living project in the Netherlands [41] used a comparable intervention approach aimed at increasing physical activity behavior and promoting healthy nutrition behavior in primary schools. Of these intervention studies, the ones that evaluated intervention effects after 2 years found positive effects on the mean steps per day on schooldays and weekends [70], fruit and vegetable consumption, caloric intake, self-reported PA levels and percentage of obese children [69]. In contrast, the interventions that were evaluated after 1 year (11 months to 16 months) found no effect on SB and PA [41, 71], BMI standard deviation score [71] or they found partial effects (i.e., an increase in average daily steps in boys, but not in girls) [68]. These results confirm that, although the implementation of a context-based, flexible intervention using a participatory approach requires a substantial amount of time to achieve effects, in the long-run they have the potential to be effective on children’s weight status, PA levels and even nutrition behavior.

Adaptation and contextual fit are fundaments of a comprehensive approach [72]. Without contextual fit, an intervention is less appropriate and thus less likely to be implemented in the long-term and to eventually become embedded into current practices [73]. The importance of long-term interventions that are contextually appropriate and that implement a comprehensive approach has been stressed in many systematic reviews on school-based interventions [16, 30, 31, 57, 74]. However, there are few school-based interventions in which working groups or action teams are responsible for development and implementation compared to the number of school-based interventions in which a prepackaged program is implemented [31].

### Strengths and limitations

We consider the quasi-experimental study design a strength of the study. It enabled us to implement our intervention in the ‘real world’ setting, which enhances the generalizability of our results [60]. However, it should be mentioned that this design had its limitations, which might increase the risk of bias. Only three control schools were included in the study compared to eight intervention schools. Besides, these control schools actively implemented a nutrition-promoting intervention. The recruitment of control schools was very challenging: schools experienced a lot of time pressure, and participation as control school in the study was considered something additional that does not necessarily benefit the children nor the school. Moreover, the intervention group differed significantly from the control group on ethnicity, parental educational level and parental age. Other studies have shown that demographics, such as educational level and ethnicity, are associated with our outcome measures [8, 9, 75–77], and thus might have influenced our results. Another limitation of this design was the difference between the intervention and control group in children’s MVPA and the percentage children consuming fruit and vegetables at school at baseline.

Moreover, dropout rates might have influenced the results of this study. At both intervention and control schools, multiple children moved to another school during the study period. Missing data were not only the result of children dropping out, but also due to children being ill during the day of measurement and/or low adherence to the accelerometer. The latter was also seen in comparable intervention studies and can be dealt with by oversampling at baseline [78].

There are also some strengths and limitations related to the study methodology. Methodological strengths of this study were the objective assessment of BMI z-score and device-based assessment of PA outcomes and the measure of both BMI z-score and the energy balance-related behaviors of children. However, it must be acknowledged that device-based measures do have some limitations. The ActiGraph accelerometers are unable to accurately measure PA associated with non-ambulatory activities like cycling [79], and we did not measure PA during activities involving water (e.g., swimming). Finally, results on SB and PA were limited to children with at least 2 days of valid data recording during school days as adherence to the wear protocol was low during weekend days. Yet, the content of the intervention was mostly designed to influence SB and PA during school days rather than weekends, lowering the impact of this limitation.

Other methodological limitations of our study are the use of self-reporting instruments for children’s nutrition behavior. These reporting instruments might lead to socially desirable answers [80] and, although the child questionnaire had a high response rate, the parent questionnaire was subject to a lower response rate (± 62%) and a selective sample (parents who had filled in the questionnaire were more likely to be of Western origin, *χ*^*2*^ (1, *N* = 520) = 41.60, *p* < .001).

A strength related to the study objectives is the link between intervention comprehensiveness and intervention effectiveness. There are limited school-based intervention studies, including studies that adopted a comprehensive school health approach, that link the level of implementation at a school level to outcomes [66]. We encourage researchers to include this objective when studying intervention effects.

## Conclusions

The KEIGAAF intervention, a mutual adaptation PA and nutrition intervention, is effective in improving children’s BMI z-score, as well as MVPA during school days. Larger effects were found on BMI z-score and PA levels when schools implemented a comprehensive PA-promoting approach. Schools with the most vulnerable population and in need for improvement in SB and PA behavior succeeded in implementing such an approach. We emphasize the importance of implementing a long-term, locally appropriate, comprehensive approach to promote a healthy weight status, to stimulate children’s PA levels, and to prevent them spending excessive time in sedentary behaviors during school days.

## Supplementary information

**Additional file 1 **Intervention schools’ level of comprehensiveness concerning physical activity promotion and healthy nutrition promotion. **Table S1.** Schools’ physical activity promotion at the end of the intervention period. **Table S2.** Schools’ healthy nutrition promotion at the end of the intervention period.

**Additional file 2 **Intervention effects of comprehensive physical activity (PA) promoting approach on children’s BMI z-score, PA levels and nutrition behavior. **Table S1.** One- and two-year observed changes in BMI z-score for (1) the comprehensive PA intervention group, (2) the less comprehensive PA intervention group and (3) the control group (Model 1) and intervention effects after one and two years comparing (1), (2) and (3) (Model 2). **Table S2.** One- and two-year observed changes in sedentary and physical activity (PA) behavior for (1) the comprehensive PA intervention group, (2) the less comprehensive PA intervention group and (3) the control group (Model 1) and intervention effects after one and two years comparing (1), (2) and (3) (Model 2). **Table S3.** One- and two-year observed changes in (child-reported) nutrition behavior at school for (1) the comprehensive physical activity (PA) intervention group, (2) the less comprehensive PA intervention group and (3) the control group (Model 1) and intervention effects after one and two years comparing (1), (2) and (3) (Model 2). **Table S4.** One- and two-year observed changes in (parent-reported) daily nutrition behavior for (1) the comprehensive physical activity (PA) intervention group, (2) the less comprehensive PA intervention group and (3) the control group (Model 1) and intervention effects after one and two years comparing (1), (2) and (3) (Model 2).

**Additional file 3 The TIDieR Checklist.**

## Data Availability

The data that support the findings of this study are available upon reasonable request from the corresponding author S.R.B. V.-J. The data are not publicly available due to them containing information that could compromise research participant privacy/consent.
