# Supplementary material for: Effects of the KEIGAAF intervention on the BMI z-score and energy balance-related behaviors of primary school-aged children
Source: Int J Behav Nutr Phys Act. 2020 Aug 17;17:105. doi: 10.1186/s12966-020-01012-8 (PMC7433155; doi:10.1186/s12966-020-01012-8)
Supplement: Supplementary file 1 — Additional file 1 Intervention schools’ level of comprehensiveness concerning physical activity promotion and healthy nutrition promotion. Table S1. Schools’ physical activity promotion at the end of the intervention period. Table S2. Schools’ healthy nutrition promotion at the end of the intervention period. [file 12966_2020_1012_MOESM1_ESM.docx]

**Additional file 1. Intervention schools’ level of comprehensiveness concerning physical activity promotion and healthy nutrition promotion**

**Table 1. Schools’ physical activity promotion at the end of the intervention period.**

|  | **School 1** | **School 2** | **School 3** | **School 4** | **School 5** | **School 6** | **School 7** | **School 8** |
| --- | --- | --- | --- | --- | --- | --- | --- | --- |
|  | **(1) Physical education** | | | | | | | |
| Weekly physical education (PE) (provided by PE-teacher and school teacher) | 60 minutes (PE-teacher) | 2x 45 minutes | 2x 45 minutes | 60 + 45 minutes | 2x 45 minutes | 50 + 45 minutes | 2x 45 minutes | 2x 45 minutes |
| Sports clinics during PE°  *(e.g., boxing clinic)* |  | ✔ | ✔ | ✔ |  | ✔ | ✔ | ✔ |
|  | **(2) Physical activity (PA) during school** | | | | | | | |
| PA activities during recess°  *(e.g., sports activity, such as basketball, organized by local social work organization)* | Three times a week | Once a week | Once a week | Once a week |  | Once a week | Four times a week | Once a week |
| PA equipment during recess  *(e.g., balls, hockey sticks, cones)* | ✔ | ✔ | ✔ | ✔ | ✔ | ✔ | ✔ | ✔ |
| Active curriculum  *(e.g., learning mathematics while being active)* | ✔ | ✔ | ✔ | ✔ |  | ✔ | ✔ | ✔ |
| Standing learning  *(e.g., standing desk for the children)* |  |  |  |  |  | ✔ | ✔ |  |
| Activity breaks  *(i.e., short bouts of activities in between lessons)* | ✔ | ✔ | ✔ | ✔ | ✔ | ✔ |  |  |
| Sports day once a year°  *(e.g., school day full of sports and play at nearby soccer field)* | ✔ | ✔ | ✔ | ✔ | ✔ | ✔ | ✔ | ✔ |
| PA and healthy nutrition week once a year  *(e.g., a whole school week full of extra PA and healthy nutrition-related activities)* |  |  |  | ✔ |  |  |  |  |
| One mile run once a week *(e.g., children run one mile around the school building in between lessons)* |  |  |  | ✔ |  |  |  |  |
|  | **(3) PA before and after school** | | | | | | | |
| After-school PA  (*i.e., after school activities, such as tag, dodgeball, soccer, organized by PE-teacher for interested children/ children of a specific age group)* | Once a month | Once a week° | Once a month | Once a month |  | Once a month | Once a week° | Once a month |
| Active transport activity once a month  *(e.g., children come to school with a bicycle, step, or roller skates and are allowed to use these during recess.)* | ✔ |  | ✔ |  |  |  |  |  |
| National sports week once a year°  *(i.e., local sport clubs provide introduction lessons free of charge. Schools promote this week under school hours.)* |  | ✔ | ✔ | ✔ |  |  | ✔ |  |
| National outdoor play day once a year  *(i.e., schools promote that children play outside after school time during this day)* |  | ✔ |  |  |  |  |  |  |
| Active transport week once a year  *(e.g., children travel to school actively. School stimulate active transport by providing activities like traffic lessons.)* | ✔ |  |  |  |  | ✔ |  |  |
| Local ice-skating event once a year°  *(i.e., interested children participate in an ice-skating competition under guidance of schoolteachers and parents)* |  | ✔ | ✔ | ✔ |  | ✔ |  | ✔ |
| Local marathon once a year°  *(i.e., interested children participate in the mini marathon or kids runs under guidance of schoolteachers and parents)* |  | ✔ | ✔ | ✔ | ✔ | ✔ | ✔ | ✔ |
| School soccer tournament once a year  *(e.g., schools organize a soccer tournament after school hours for interested children)* |  |  |  |  |  | ✔ |  |  |
| Three-day neighborhood walk once a year°  *(e.g., interested children participate in a neighborhood walk after school hours for three days in a row under guidance of parents)* |  |  |  |  |  |  | ✔ | ✔ |
|  | **(4) PA policy** | | | | | | | |
| PA policy | Written policy | Unwritten policy | Unwritten policy | Written policy | Written policy | No policy | Unwritten policy | Unwritten policy |
| Support of PA policy by school staff | High | Very high | Very high | Very high | High | NA | High | Very high |
|  | **(5) Staff involvement in school PA policy/activities, degree of …** | | | | | | | |
| … stimulation of PA activities during recess | High | Moderate | High | Moderate | Moderate | Moderate | High | Low |
| … active transport stimulation | High | Moderate | High | Very high | High | Moderate | Moderate | Low |
| … implementation of school PA policy | High | Moderate | High | Moderate | Moderate | NA | High | Moderate |
|  | **(6) Parental engagement in school PA activities, parental engagement in …** | | | | | | | |
| … activities during recess |  |  |  |  |  |  |  |  |
| … implementing sports days | ✔ | ✔ | ✔ | ✔ | ✔ | ✔ | ✔ | ✔ |
| … active transport stimulation | ✔ |  | ✔ | ✔ |  |  |  |  |
| … after school PA |  |  |  |  |  | ✔ |  |  |
|  | **Level of PA comprehensiveness*** | | | | | | | |
| Comprehensiveness | LOW | HIGH | LOW | HIGH | LOW | HIGH | HIGH | LOW |

*Note.* Activities and actions in green were implemented or enhanced/improved during the intervention period (activities and actions in black were already present at the start of the intervention). The provided examples may differ per school and are merely provided to give an idea of the implemented activities.

* Comparison of comprehensiveness of PA promotion between schools: low versus high. Level of comprehensiveness was based on activities implemented in all categories (presented in the table), coherence between practice and policies and a consistent spread of the message via different channels inside and outside the school environment.

° Activity was organized by an external organization, e.g. local sports club, municipal sports support organization, or event organizers.

**Table 2. Schools’ healthy nutrition promotion at the end of the intervention period.**

|  | **School 1** | **School 2** | **School 3** | **School 4** | **School 5** | **School 6** | **School 7** | **School 8** |
| --- | --- | --- | --- | --- | --- | --- | --- | --- |
|  | **(1) Nutrition education** | | | | | | | |
| Educational program “Nice and Fit”°  *(i.e., education on nutrition, PA and making healthy choices for all grades consisting of 10 educational hours)* | ✔ |  | ✔ | ✔ | ✔ |  | ✔ | ✔ |
| “Taste lessons” / “Taste Mission”°  *(i.e., education on nutrition for all grades. In five lessons (of 60 minutes each) children experiment with nutrition.)* | ✔ | ✔ | ✔ | ✔ |  | ✔ |  |  |
| “I eat it better” lessons°  *(i.e., education on nutrition for the higher grades (children aged 9 to 12 years) consisting of two hours)* |  | ✔ | ✔ | ✔ |  | ✔ |  |  |
| EU school fruit lessons°  *(i.e., education on fruit and vegetables for all grades consisting of five to eight lessons (of 50-60 minutes each)* | ✔ | ✔ |  | ✔ | ✔ | ✔ | ✔ | ✔ |
| National tap water day once a year°  *(i.e., schools educate children on the importance of consuming tap water)* |  |  |  |  | ✔ | ✔ |  |  |
| National Breakfast event lessons°  *(i.e., animation movies and one lesson educating on the importance of a healthy breakfast for all grades)* |  |  |  |  |  | ✔ |  |  |
|  | **(2) Healthy nutrition during school** | | | | | | | |
| EU school fruit for 20 weeks°  *(i.e., 3 pieces of fruit per child per week)* | ✔ | ✔ |  | ✔ | ✔ | ✔ | ✔ | ✔ |
| Vegetable garden  *(e.g., children take care of the vegetable garden)* |  |  |  |  | ✔ |  | ✔ |  |
| Water bottles/water jugs (daily)  *(e.g., each child has a water bottle at school which has to be filled with water)* | ✔ | ✔ | ✔ |  |  | ✔ |  | ✔ |
| Healthy breakfast week once a year  *(e.g., children consume a healthy breakfast at school during a whole week)* | ✔ |  |  |  |  |  |  |  |
| National breakfast activity°  *(e.g., children consume a healthy breakfast at school)* | Twice a year | Twice a year | Once a year | Twice a year | Three times a year | Twice a year |  | Twice a year |
| Healthy nutrition week once a year  *(e.g., attention is paid to healthy nutrition during a whole week by having healthy lunch competitions, and reminding the children and parents of the nutrition rules)* | ✔ |  |  | ✔ |  |  |  |  |
| National healthy snack week once a year°  *(i.e., schools pay attention to healthy snacks by showing movies, providing healthy snacks and challenging children to perform certain healthy snack related tasks)* |  | ✔ | ✔ | ✔ |  | ✔ |  |  |
| Water promotion week once a year  *(e.g., children are requested and challenged to only drink water for a whole week)* |  |  |  | ✔ |  |  |  |  |
| Fruit promotion month once a year  *(e.g., children are challenged to consume fruit instead of unhealthy snacks during recess by participating in a fruit competition)* |  |  |  |  |  | ✔ |  |  |
| Healthy birthday treats session once year°  *(e.g., the local municipal health service provides a workshop for all schoolchildren on making healthy birthday treats)* |  |  |  |  |  | ✔ |  |  |
| Cooking workshop once a year  *(e.g., interested children attend a workshop on cooking)* |  |  |  |  |  |  | ✔ |  |
|  | **(3) Healthy nutrition before and after school** | | | | | | | |
| Cooking workshop (10 lessons) twice a year°  *(e.g., interested children participate in a workshop on cooking organized by a social organization)* | ✔ |  |  |  |  |  |  |  |
| Healthy nutrition and birthday treats at out-of-school care center (continuous)°  *(e.g., birthday children make their own healthy birthday treats at the out-of-school care center)* |  |  |  | ✔ |  |  |  |  |
| Water tap at nearby playground°  *(e.g., a water tap is placed at a public playground by the municipality)* |  |  |  |  | ✔ |  |  |  |
|  | **(4) Nutrition policy** | | | | | | | |
| Nutrition policy | Written nutrition policy | Written nutrition policy | Written nutrition policy | Written nutrition policy | Unwritten nutrition policy | Written nutrition policy | Written nutrition policy | Written nutrition policy |
| Nutrition policy concerning… |  |  |  |  |  |  |  |  |
| … healthy lunch | ✔ | ✔ | ✔ | ✔ | ✔ | ✔ | ✔ |  |
| … healthy drinks (i.e., water) | ✔ | ✔ | ✔ | ✔ | ✔ | ✔ | ✔ | ✔ |
| … healthy birthday treats | ✔ |  | ✔ | ✔ |  | ✔ | ✔ | ✔ |
| … healthy snacks (i.e., fruit and vegetables) | ✔ | ✔ | ✔ | ✔ |  | ✔ | ✔ | ✔ |
| … modeling healthy nutrition behavior by teachers | ✔ | ✔ | ✔ | ✔ |  | ✔ |  | ✔ |
| Support of nutrition policy by school staff | High | Moderate | High | High | Moderate | High | High | High |
|  | **(5) Staff involvement in school nutrition policy/activities, degree of …** | | | | | | | |
| Degree of implementation of school nutrition policies | High | Moderate | High | High | Moderate | High | High | High |
|  | **(6) Parental engagement in school nutrition activities, parental engagement in …** | | | | | | | |
| … school nutrition education | ✔ |  |  | ✔ | ✔ |  |  |  |
| … nutrition policy | ✔ | ✔ |  | ✔ |  | ✔ |  | ✔ |
| … nutrition activities | ✔ |  |  | ✔ |  |  | ✔ |  |

*Note.* Activities and actions in green were implemented or enhanced/improved during the intervention period (activities and actions in black were already present at the start of the intervention). The provided examples may differ per school and are merely provided to give an idea of the implemented activities.

° Activity was developed and/or organized by an external organization, e.g. the Dutch Nutrition Centre, local social work organization, local cooking organization.
