# Supplementary material for: Effects of the KEIGAAF intervention on the BMI z-score and energy balance-related behaviors of primary school-aged children
Source: Int J Behav Nutr Phys Act. 2020 Aug 17;17:105. doi: 10.1186/s12966-020-01012-8 (PMC7433155; doi:10.1186/s12966-020-01012-8)
Supplement: Supplementary file 2 — Additional file 2 Intervention effects of comprehensive physical activity (PA) promoting approach on children’s BMI z-score, PA levels and nutrition behavior. Table S1. One- and two-year observed changes in BMI z-score for (1) the comprehensive PA intervention group, (2) the less comprehensive PA intervention group and (3) the control group (Model 1) and intervention effects after one and two years comparing (1), (2) and (3) (Model 2). Table S2. One- and two-year observed changes in sedentary and physical activity (PA) behavior for (1) the comprehensive PA intervention group, (2) the less comprehensive PA intervention group and (3) the control group (Model 1) and intervention effects after one and two years comparing (1), (2) and (3) (Model 2). Table S3. One- and two-year observed changes in (child-reported) nutrition behavior at school for (1) the comprehensive physical activity (PA) intervention group, (2) the less comprehensive PA intervention group and (3) the control group (Model 1) and intervention effects after one and two years comparing (1), (2) and (3) (Model 2). Table S4. One- and two-year observed changes in (parent-reported) daily nutrition behavior for (1) the comprehensive physical activity (PA) intervention group, (2) the less comprehensive PA intervention group and (3) the control group (Model 1) and intervention effects after one and two years comparing (1), (2) and (3) (Model 2). [file 12966_2020_1012_MOESM2_ESM.docx]

**Additional file 2. Intervention effects of comprehensive physical activity (PA) promoting approach on children’s BMI z-score, PA levels and nutrition behavior**

**Table 1. One- and two-year observed changes in BMI z-score for (1) the comprehensive PA intervention group, (2) the less comprehensive PA intervention group and (3) the control group (Model 1) and intervention effects after one and two years comparing (1), (2) and (3) (Model 2).**

|  |  | **Model 1** | | | | | | | **Model 2** | | | | | | | | | |
| --- | --- | --- | --- | --- | --- | --- | --- | --- | --- | --- | --- | --- | --- | --- | --- | --- | --- | --- |
|  |  | **(1) (N = 141)** | | **(2) (N = 278)** | | **(3) (N = 104)** | | | **(1) vs (3)** | | | **(2) vs (3)** | | | **(1) vs (2)** | | |  |
|  |  | **N** | **Mean (SD)^a^** | **N** | **Mean (SD)^a^** | | **N** | **Mean (SD)^a^** | **B (95% CI)^b^** | **p** | **ES^c^** | **B (95% CI)^b^** | **p** | **ES^c^** | **B (95% CI)^b^** | **p** | **ES^c^** |  |
| BMI z- | T0 | 133 | 0.38 (1.08) | 270 | 0.15 (1.09) | | 98 | 0.22 (0.97) | Ref1 |  |  | Ref1 |  |  | Ref2 |  |  |  |
| score | T1 | 130 | 0.39 (1.03) | 253 | 0.23 (1.13) | | 91 | 0.38 (1.03) | **-0.17 (-0.29; -0.05)** | **0.01** | **-0.07** | **-0.08 (-0.19; 0.03)** | **0.00** | -0.14 | -0.09 (-0.19; 0.00) | 0.06 | -0.01 |  |
|  | T2 | 117 | 0.24 (1.01) | 240 | 0.15 (1.07) | | 83 | 0.38 (1.06) | **-0.35 (-0.50; -0.19)** | **0.00** | **-0.06** | -0.20 (-0.34; -0.07) | 0.16 | -0.10 | **-0.15 (-0.27; -0.02)** | **0.02** | **-0.21** |  |

*Note.* Analyses were conducted with repeated measures linear mixed model analysis with adjustment for clustering of data within persons. Predictor variable is time*condition (Ref1 = baseline measurement*control; Ref2 = baseline measurement*less comprehensive PA promoting approach). Analyses were adjusted for ethnicity (Western vs. non-Western), and the residential socioeconomic status score

(1) comprehensive PA promoting approach, (2) less comprehensive PA promoting approach, (3) control group, BMI = Body Mass Index, T0 = baseline measurement, T1 = measurement at year one, T2 = measurement at year two, SD = standard deviation, B = unstandardized beta coefficient, CI = confidence interval, p = p-value, ES = effect size, Ref = reference.

Bold numbers are significant at p < 0.05.

^a^ Mean is the observed BMI z-score of participants with data.

^b^ Unstandardized beta coefficient of linear mixed models.

^c^ Cohen’s d effect size, calculated with estimated means of linear mixed models.

**Table 2. One- and two-year observed changes in sedentary and physical activity (PA) behavior for (1) the comprehensive PA intervention group, (2) the less comprehensive PA intervention group and (3) the control group (Model 1) and intervention effects after one and two years comparing (1), (2) and (3) (Model 2).**

|  |  | **Model 1** | | | | | | | **Model 2** | | | | | | | | | |
| --- | --- | --- | --- | --- | --- | --- | --- | --- | --- | --- | --- | --- | --- | --- | --- | --- | --- | --- |
|  |  | **(1) (N = 141)** | | **(2) (N = 278)** | | **(3) (N = 104)** | | | **(1) vs (3)** | | | **(2) vs (3)** | | | **(1) vs (2)** | | |  |
|  |  | **N** | **Mean (SD)^a^** | **N** | **Mean (SD)^a^** | | **N** | **Mean (SD)^a^** | **B (95% CI)^b^** | **p** | **ES^c^** | **B (95% CI)^b^** | **p** | **ES^c^** | **B (95% CI)^b^** | **p** | **ES^c^** |  |
| SB (%) | T0 | 118 | 63.28 (6.23)* | 261 | 60.46 (6.56) | | 84 | 60.03 (7.11) | Ref1 |  |  | Ref1 |  |  | Ref2 |  |  |  |
|  | T1 | 99 | 65.83 (5.57) | 227 | 63.86 (6.64) | | 75 | 62.66 (6.95) | -0.18 (-1.96; 1.61) | 0.85 | -0.02 | 0.10 (-1.50; 1.71) | 0.90 | 0.01 | -0.28 (-1.85; 1.30) | 0.73 | -0.02 |  |
|  | T2 | 89 | 65.48 (7.12) | 176 | 67.25 (5.94) | | 67 | 66.39 (6.83) | **-2.89 (-5.05; -0.74)** | **0.01** | **-0.31** | 0.44 (-1.30; 2.19) | 0.62 | 0.04 | **-3.34 (-4.98; 1.70)** | **0.00** | **-0.23** |  |
| LPA | T0 | 118 | 29.44 (5.08)* | 261 | 31.52 (4.94) | | 84 | 31.34 (5.36) | Ref1 |  |  | Ref1 |  |  | Ref2 |  |  |  |
| (%) | T1 | 99 | 27.37 (4.64) | 227 | 28.85 (4.97) | | 75 | 29.11 (5.13) | 0.05 (-1.35; 1.45) | 0.95 | 0.01 | 0.11 (-1.15; 1.38) | 0.86 | 0.01 | -0.07 (-1.30; 1.16) | 0.91 | -0.01 |  |
|  | T2 | 89 | 27.20 (5.45) | 176 | 26.18 (4.37) | | 67 | 27.01 (5.27) | 0.96 (-0.70; 2.61) | 0.26 | 0.14 | -1.14 (-2.49; 0.22) | 0.10 | -0.14 | **2.09 (0.83; 3.36)** | **0.00** | **0.19** |  |
| MVPA | T0 | 118 | 7.27 (2.83)† | 261 | 8.02 (2.89) | | 84 | 8.63 (3.78) | Ref1 |  |  | Ref1 |  |  | Ref2 |  |  |  |
| (%) | T1 | 99 | 6.80 (2.31) | 227 | 7.29 (2.69) | | 75 | 8.23 (3.18) | 0.03 (-0.79; 0.86) | 0.94 | 0.00 | -0.20 (-0.95; 0.54) | 0.59 | -0.04 | 0.24 (-0.49; 0.96) | 0.52 | 0.04 |  |
|  | T2 | 89 | 7.32 (3.28) | 176 | 6.57 (2.52) | | 67 | 6.60 (2.34) | **1.84 (0.82; 2.85)** | **0.00** | **0.44** | 0.65 (-0.18; 1.48) | 0.13 | 0.13 | **1.19 (0.42; 1.96)** | **0.00** | **0.18** |  |
| CPM | T0 | 118 | 1085.00 (270.36)* | 261 | 1185.04 (291.89) | | 84 | 1270.87 (489.96) | Ref1 |  |  | Ref1 |  |  | Ref2 |  |  |  |
| (%) | T1 | 99 | 1000.75 (238.79) | 227 | 1058.15 (266.52) | | 75 | 1131.00 (323.44) | 56.38 (-33.74; 146.49) | 0.22 | 0.12 | 16.13 (-65.19; 97.44) | 0.70 | 0.03 | 40.25 (-38.31; 118.82) | 0.32 | 0.05 |  |
|  | T2 | 89 | 1013.55 (298.68) | 176 | 951.11 (273.80) | | 67 | 926.21 (227.56) | **225.52 (118.02; 333.02)** | **0.00** | **0.48** | **97.48 (8.82; 186.13)** | **0.03** | **0.18** | **128.04 (45.54; 210.54)** | **0.00** | **0.17** |  |

*Note.* Analyses were conducted with repeated measures linear mixed model analysis with adjustment for clustering of data within persons and adjustment of clustering of data at school level. Predictor variable is time*condition (Ref1 = baseline measurement*control; Ref2= baseline measurement*less comprehensive PA promoting approach). Analyses were adjusted for children’s age at baseline, BMI z-score at baseline, gender, ethnicity (Western vs. non-Western), the residential socioeconomic status score and weather conditions (i.e., sunshine, temperature and precipitation).

(1) comprehensive PA promoting approach, (2) less comprehensive PA promoting approach, (3) control group, SB = Sedentary behavior, LPA = Light physical activity behavior, MVPA = moderate-to-vigorous physical activity behavior, CPM = counts per minute, T0 = baseline measurement, T1 = measurement at year one, T2 = measurement at year two, SD = standard deviation, B = unstandardized beta coefficient, CI = confidence interval, p = p-value, ES = effect size, Ref = reference.

Bold numbers are significant at p < 0.05.

* Significantly different from the baseline levels of the children of the less comprehensive PA intervention group and the children of the control group (Welch’s test and Games-Howell post-hoc analysis).

† Significantly different from the baseline levels of the children of the control group, but not compared to the children of the less comprehensive PA intervention group (Welch’s test and Games-Howell post-hoc analysis).

^a^ Mean is the observed SB, LPA, MVPA and CPM of participants with data.

^b^ Unstandardized beta coefficient of linear mixed models.

^c^ Cohen’s d effect size, calculated with estimated means of linear mixed models.

**Table 3. One- and two-year observed changes in (child-reported) nutrition behavior at school for (1) the comprehensive physical activity (PA) intervention group, (2) the less comprehensive PA intervention group and (3) the control group (Model 1) and intervention effects after one and two years comparing (1), (2) and (3) (Model 2).**

|  |  | **Model 1** | | | | | | | **Model 2** | | | | | | |
| --- | --- | --- | --- | --- | --- | --- | --- | --- | --- | --- | --- | --- | --- | --- | --- |
|  |  | **(1) (N = 141)** | | **(2) (N = 278)** | | **(3) (N = 104)** | | **(1) vs (3)** | | | **(2) vs (3)** | | **(1) vs. (2)** | |  |
|  |  | **N** | **Obs %^a^** | **N** | **Obs %^a^** | **N** | **Obs %^a^** | **OR (95% CI)^b^** | | **p** | **OR (95%CI)^b^** | **p** | **OR (95%CI)^b^** | **p** |  |
| Breakfast (%yes) | T0 | 141 | 91.5 | 272 | 93.8 | 101 | 87.1 | Ref1 | |  | Ref1 |  | Ref2 |  |  |
|  | T1 | 126 | 88.9 | 250 | 90.8 | 90 | 76.7 | 1.85 (0.64; 5.38) | | 0.26 | 1.34 (0.52; 3.52) | 0.55 | 1.38 (0.52; 3.68) | 0.52 |  |
|  | T2 | 117 | 88.0 | 236 | 89.4 | 81 | 75.3 | 1.99 (0.68; 5.83) | | 0.21 | 1.99 (0.68; 5.83) | 0.41 | 1.33 (0.50; 3.55) | 0.57 |  |
| Fruit or vegetables (%yes) | T0 | 141 | 88.7† | 271 | 89.7 | 101 | 75.2 | Ref1 | |  | Ref1 |  | Ref2 |  |  |
|  | T1 | 126 | 92.1 | 249 | 88.8 | 90 | 75.6 | 2.16 (0.82; 5.68) | | 0.19 | 1.22 (0.54; 2.72) | 0.63 | 1.78 (0.72; 4.38) | 0.21 |  |
|  | T2 | 116 | 90.5 | 236 | 91.1 | 81 | 74.1 | 2.01 (0.75; 5.37) | | 0.17 | 1.55 (0.67; 3.59) | 0.31 | 1.30 (0.51; 3.28) | 0.58 |  |
| Candy, cookies or snacks | T0 | 141 | 41.1 | 271 | 44.3 | 101 | 42.6 | Ref1 | |  | Ref1 |  | Ref2 |  |  |
| (%yes) | T1 | 126 | 30.2 | 250 | 48.0 | 90 | 35.6 | 0.80 (0.39; 1.67) | | 0.56 | 1.60 (0.85; 3.02) | 0.15 | **0.50 (0.28; 0.89)** | **0.02** |  |
|  | T2 | 116 | 28.4 | 237 | 41.4 | 81 | 38.3 | 0.64 (0.30; 1.35) | | 0.24 | 1.03 (0.54; 1.96) | 0.94 | 0.62 (0.34; 1.13) | 0.12 |  |
| Sugar-sweetened | T0 | 141 | 61.0* | 271 | 50.6 | 101 | 47.5 | Ref1 | |  | Ref1 |  | Ref2 |  |  |
| beverages (%yes) | T1 | 126 | 47.6 | 250 | 51.2 | 90 | 13.3 | **3.35 (1.54; 7.252)** | | **0.00** | **5.85 (2.88; 11.90)** | **0.00** | **0.57 (0.34; 0.97)** | **0.04** |  |
|  | T2 | 116 | 45.7 | 236 | 34.3 | 81 | 7.4 | **5.94 (2.31; 15.27)** | | **0.00** | **5.51 (2.26; 13.45)** | **0.00** | 1.08 (0.63; 1.86) | 0.79 |  |
| Water (%yes) | T0 | 141 | 52.5 | 267 | 60.3 | 101 | 59.4 | Ref1 | |  | Ref1 |  | Ref2 |  |  |
|  | T1 | 126 | 61.9 | 250 | 63.6 | 90 | 95.6 | **0.09 (0.03; 0.25)** | | **0.00** | **0.08 (0.03; 0.21)** | **0.00** | 1.14 (0.67; 1.92) | 0.63 |  |
|  | T2 | 116 | 66.4 | 236 | 74.2 | 81 | 86.4 | **0.37 (0.17; 0.83)** | | **0.02** | **0.39 (0.19; 0.83)** | **0.02** | 0.95 (0.54; 1.66) | 0.86 |  |

*Note.* Analyses were conducted with generalized estimating equations analysis with adjustment for clustering of data within persons. Predictor variable is time*condition (Ref1 = baseline measurement*control; Ref2 = baseline measurement*less comprehensive PA promoting approach). Analyses were adjusted for children’s age at baseline, BMI z-score at baseline, gender, ethnicity (Western vs. non-Western), and the residential socioeconomic status score.

(1) comprehensive PA promoting approach, (2) less comprehensive PA promoting approach, (3) control group, T0 = baseline measurement, T1 = measurement at year one, T2 = measurement at year two, Obs % = observed percentage, OR = odds ratio, CI = confidence interval, p = p-value, ES = effect size, Ref = reference.

Bold numbers are significant at p < 0.05.

* Significantly different from the baseline levels of the children of the less comprehensive PA intervention group and the children of the control group (Pearson Chi-Square test).

† Significantly different from the baseline levels of the children of the control group, but not compared to the children of the less comprehensive PA intervention group (Pearson Chi-Square test).

^a^ Mean is the observed percentage of children consuming the food/drink.

^b^ Odds ratio of GEE model adjusted for child age, gender, ethnicity and BMI z-score at baseline and residential socioeconomic status score at baseline.

**Table 4. One- and two-year observed changes in (parent-reported) daily nutrition behavior for (1) the comprehensive physical activity (PA) intervention group, (2) the less comprehensive PA intervention group and (3) the control group (Model 1) and intervention effects after one and two years comparing (1), (2) and (3) (Model 2).**

|  |  | **Model 1** | | | | | | **Model 2** | | | | | |
| --- | --- | --- | --- | --- | --- | --- | --- | --- | --- | --- | --- | --- | --- |
|  |  | **(1) (N = 141)** | | **(2) (N = 278)** | | **(3) (N = 104)** | | **(1) vs (3)** | | **(2) vs (3)** | | **(1) vs (2)** | |
|  |  | **N** | **Obs %^a^** | **N** | **Obs %^a^** | **N** | **Obs %^a^** | **OR (95% CI)^b^** | **p** | **OR (95%CI)^b^** | **p** | **OR (95%CI)^b^** | **p** |
| Adherence fruit | T0 | 87 | 32.2 | 176 | 41.5 | 48 | 27.1 | Ref1 |  | Ref1 |  | Ref2 |  |
| recommendation | T1 | 77 | 44.2 | 165 | 41.8 | 57 | 22.8 | 1.78 (0.67; 4.77) | 0.25 | 1.17 (0.48; 2.85) | 0.73 | 1.52 (0.77; 3.02) | 0.23 |
| (%yes) | T2 | 81 | 38.3 | 171 | 33.9 | 58 | 20.7 | 1.98 (0.72; 5.41) | 0.19 | 0.97 (0.39; 2.42) | 0.95 | **2.04 (1.02; 4.10)** | **0.05** |
| Adherence vegetable | T0 | 79 | 24.1 | 171 | 26.9 | 52 | 23.1 | Ref1 |  | Ref1 |  | Ref2 |  |
| recommendation | T1 | 72 | 16.7 | 161 | 25.5 | 58 | 12.1 | 1.49 (0.42; 5.30) | 0.54 | 1.95 (0.64; 5.97) | 0.24 | 0.76 (0.31; 1.89) | 0.56 |
| (%yes) | T2 | 78 | 10.3 | 161 | 20.5 | 54 | 3.7 | 2.62 (0.49; 14.11) | 0.26 | **5.42 (1.18; 24.98)** | **0.03** | 0.48 (0.18; 1.32) | 0.16 |
| Daily consumption of | T0 | 87 | 37.9† | 187 | 50.8 | 52 | 50.0 | Ref1 |  | Ref1 |  | Ref2 |  |
| Snacks (%yes) | T1 | 82 | 22.0 | 173 | 35.3 | 61 | 44.3 | 0.56 (0.25; 1.27) | 0.17 | 0.61 (0.31; 1.22) | 0.17 | 0.92 (0.48; 1.75) | 0.80 |
|  | T2 | 86 | 29.1 | 178 | 37.1 | 63 | 28.6 | 1.70 (0.75; 3.84) | 0.20 | 1.54 (0.75; 3.16) | 0.24 | 1.11 (0.60; 2.04) | 0.75 |
| Daily consumption of | T0 | 87 | 66.7† | 183 | 50.8 | 51 | 52.9 | Ref1 |  | Ref1 |  | Ref2 |  |
| sugar-sweetened | T1 | 80 | 53.8 | 169 | 39.1 | 62 | 59.7 | **0.42 (0.18; 0.98)** | **0.04** | **0.46 (0.22; 0.98)** | **0.04** | 0.90 (0.46; 1.76) | 0.77 |
| beverages (%yes) | T2 | 85 | 41.2 | 180 | 35.6 | 63 | 42.9 | 0.46 (0.20; 1.09) | 0.08 | 0.81 (0.38; 1.73) | 0.58 | 0.57 (0.29; 1.11) | 0.10 |
| Daily consumption of | T0 | 85 | 62.4 | 188 | 69.7 | 52 | 61.5 | Ref1 |  | Ref1 |  | Ref2 |  |
| Water (%yes) | T1 | 79 | 69.6 | 172 | 70.9 | 62 | 72.6 | 0.77 (0.33; 1.79) | 0.54 | 0.56 (0.27; 1.19) | 0.13 | 1.37 (0.71; 2.62) | 0.35 |
|  | T2 | 86 | 70.9 | 181 | 71.3 | 62 | 71.0 | 0.90 (0.39; 2.10) | 0.81 | 0.64 (0.30; 1.36) | 0.24 | 1.41 (0.74; 2.69) | 0.29 |

*Note.* Analyses were conducted with generalized estimating equations analysis with adjustment for clustering of data within persons. Predictor variable is time*condition (Ref1 = baseline measurement*control; Ref2 = baseline measurement*less comprehensive PA promoting approach). Analyses were adjusted for children’s age at baseline, BMI z-score at baseline, gender, ethnicity (Western vs. non-Western), and the residential socioeconomic status score.

(1) comprehensive PA promoting approach, (2) less comprehensive PA promoting approach, (3) control group, T0 = baseline measurement, T1 = measurement at year one, T2 = measurement at year two, Obs % = observed percentage, OR = odds ratio, CI = confidence interval, p = p-value, ES = effect size, Ref = reference.

Bold numbers are significant at p < 0.05.

† Significantly different from the baseline levels of the children of the control group, but not compared to the children of the less comprehensive PA intervention group (Pearson Chi-Square test).

^a^ Mean is the observed percentage of children consuming the food/drink.

^b^ Odds ratio of GEE model adjusted for child age, gender, ethnicity and BMI z-score at baseline and residential socioeconomic status score at baseline.
